# Protein Restriction Increases Soluble Leptin Receptor via a Leptin-Dependent Mechanism Without Affecting Leptin-Induced Appetite Suppression in Mice

**DOI:** 10.3390/nu18101482

**Published:** 2026-05-07

**Authors:** Mizuki Uchiyama, Tamaki Toyama, Yu Takei, Hinata Ishii, Asako Takenaka

**Affiliations:** Department of Agricultural Chemistry, School of Agriculture, Meiji University, Kawasaki 214-8571, Kanagawa, Japan

**Keywords:** protein restriction, leptin, Ob-R, soluble Ob-R, leptin receptor

## Abstract

Background/Objectives: Leptin, an adipocyte-derived hormone, suppresses appetite and regulates adiposity. Its soluble receptor (sOb-R), generated from the extracellular domain of the leptin receptor (Ob-R), circulates as a leptin-binding protein. We previously demonstrated that dietary protein restriction increased hepatic *Ob-R* mRNA expression and plasma sOb-R levels in C57BL/6J mice. However, the mechanism underlying this increase and its physiological relevance remain unclear. This study aimed to determine whether leptin is required for the protein-restriction-induced rise in circulating sOb-R and to evaluate whether elevated sOb-R modifies leptin responsiveness. Methods: C57BL/6J and *ob/ob* mice were fed a low-protein diet to assess the effects on hepatic *Ob-R* expression and plasma sOb-R levels. To examine whether increased sOb-R affects leptin action, exogenous leptin was administered to both strains under control and protein-restricted conditions, and appetite responses were evaluated. Results: Protein restriction increased hepatic *Ob-R* mRNA expression in both strains. Plasma sOb-R levels were elevated in C57BL/6J mice but not in *ob/ob* mice, indicating that endogenous leptin is essential for the protein-restriction-induced increase in circulating sOb-R. The anorexigenic effect of exogenous leptin was not altered by protein restriction in either strain. Conclusions: Protein restriction elevates circulating sOb-R through a leptin-dependent mechanism; however, this increase does not modify leptin-induced appetite suppression. These findings provide insight into the regulation of sOb-R under protein-restricted conditions.

## 1. Introduction

Leptin, a hormone secreted by adipose tissue, reduces adipose tissue mass through appetite suppression and the mobilization of stored lipids [1,2,3,4,5]. *Ob/ob* mice with a mutation in the *Leptin* gene develop obesity and are widely used in leptin and obesity research [6]. Leptin exerts its actions by stimulating intracellular Janus kinase/signal transducer and activator of transcription signaling through the cell surface leptin receptor, Ob-R [7]. In the hypothalamus, Ob-R regulates appetite by inhibiting the orexigenic neuropeptides neuropeptide Y (NPY) and agouti-related peptide (AgRP) and stimulating the production of the anorexigenic peptides pro-opiomelanocortin (POMC) and cocaine- and amphetamine-regulated transcript (CART) [8]. In addition, leptin increases energy expenditure by activating the sympathetic nervous system and exerts lipid-lowering effects [9]. Ob-R has at least six isoforms from Ob-Ra to f, which are produced by alternative splicing; the longest of them, Ob-Rb, is responsible for normal intracellular signal transduction [10,11]. The Ob-Re isoform consists only of the extracellular domain and is released extracellularly after synthesis to become the free leptin receptor sOb-R in the circulation [12].

Soluble Ob-R (sOb-R) is also generated by proteolytic cleavage of the extracellular portion of Ob-R, which contains a transmembrane domain [12]. A liver protease, a disintegrin and metalloproteinase domain 10 (ADAM10), has been reported to mediate this cleavage [13]. Blood sOb-R functions as a leptin-binding protein that inhibits leptin action by sequestering it [14,15]. The ratio of circulating free leptin to sOb-R-bound leptin, known as the free leptin index (FLI), is used as an indicator of leptin resistance [16]. In contrast, sOb-R has also been reported to enhance leptin activity by increasing its half-life in the circulation [17]. However, the mechanisms by which sOb-R regulates leptin activity remain unclear. Numerous studies have examined variations in circulating sOb-R levels in humans, showing that they are lower in individuals with obesity and higher in lean individuals, and are inversely correlated with body mass index and circulating leptin levels [18,19,20]. In addition, circulating sOb-R levels increase with food restriction and fasting in both humans and experimental animals [21,22]. Elevated hepatic *Ob-R* mRNA expression and blood sOb-R levels have also been observed in insulin receptor-knockout mice, suggesting that energy deprivation and reduced insulin signaling may contribute to increases in circulating sOb-R [23].

We previously reported that hepatic *Ob-R* mRNA and plasma sOb-R levels increased, whereas hepatic Ob-R protein levels decreased during dietary protein restriction [24]. Furthermore, we demonstrated that these changes in Ob-R expression were liver-specific [24]. Because protein restriction does not alter plasma leptin levels [24], Ob-R and sOb-R appear to be regulated independently of leptin availability. Protein restriction leads to impaired growth and loss of body protein, which are accompanied by altered plasma concentrations of insulin-like growth factor-I and its binding proteins [25,26,27,28]. Insulin secretion also decreases during protein restriction, while blood glucose levels remain normal owing to enhanced hepatic insulin signaling [29,30]. In addition, plasma fibroblast growth factor 21 levels increase under protein-deficient conditions and contribute to elevated energy expenditure [31,32]. These findings collectively indicate that the excess energy generated by reduced body protein stores is dissipated as thermogenesis in adipose tissue. Thus, protein restriction affects carbohydrate, energy, and lipid metabolism, in addition to protein metabolism, to maintain whole-body homeostasis. Consequently, it is highly likely that the increase in circulating sOb-R observed during protein restriction also plays a role in maintaining homeostasis.

This study aimed to elucidate the mechanism underlying the increase in plasma sOb-R during protein restriction and its physiological significance. Because leptin is thought to regulate circulating sOb-R levels, we first investigated the role of leptin in the protein-restriction-induced increase in sOb-R using *ob/ob* mice. We then examined whether the increase in sOb-R caused by protein restriction affects leptin action. C57BL/6J mice and *ob/ob* mice were fed either a low-protein diet or a control diet for 7 days, followed by once-daily intraperitoneal administration of leptin or vehicle from days 8 to 11. Using this approach, we assessed whether protein restriction alters the appetite-suppressing effect of exogenous leptin.

## 2. Materials and Methods

### 2.1. Animals

Four-week-old male C57BL/6J mice (*n* = 24) (Japan SLC, Inc., Shizuoka, Japan) and 7-week-old male *ob/ob* mice (C57BL/6JHamSlc-*ob/ob*) (*n* = 24) (Japan SLC, Inc.) were purchased from Sankyo Labo Service Corp. Inc., Tokyo, Japan. Mice were kept at 22–24 °C under a 12 h (06:00–18:00) light/dark cycle and allowed free access to tap water throughout the experiment. They were fed *ad libitum*, a commercial pellet feed (certified diet MF; Oriental Yeast, Tokyo, Japan), before the feeding experiment.

### 2.2. Animal Experiments

Mice were housed individually in stainless-steel cages and fed a control diet with 20% casein as a protein source (20C, Table 1) for 3 days to acclimate to the powdered feed. They were then divided into two groups, and 20C was administered to one group. In the other group, a low-protein diet with 5% casein (5C, Table 1) was administered to C57BL/6J mice, and one with 1% casein (1C, Table 1) was administered to *ob/ob* mice. The protein content of the protein-restricted diets for C57BL/6J and *ob/ob* mice was determined separately, based on preliminary experiments, at levels that induce increased *Ob-R* mRNA expression. Twelve mice were assigned to each experimental group. On days 8–11, 2 mg/kg leptin was injected intraperitoneally once a day into half the mice in each diet group, and vehicle (PBS) was injected into the other half. Five hours after the last injection, the mice were anesthetized with 1 mL/kg intraperitoneal sodium pentobarbital (Somnopentyl; Kyoritsu Seiyaku, Tokyo, Japan) for C57BL/6J mice and with isoflurane (DS Pharma Animal Health, Tokyo, Japan) for *ob/ob* mice. Heparinized blood was collected by cardiac puncture, and plasma samples were obtained. The liver, hypothalamus, inguinal WAT (iWAT), eWAT, and BAT were excised, weighed, frozen in liquid nitrogen, and stored at –80 °C until analysis.

### 2.3. RNA Preparation and Real-Time PCR

Total RNA was extracted from 50–100 mg of liver, 100–150 mg of eWAT, or the total amount of hypothalamus derived from one mouse and used for cDNA synthesis and real-time PCR using TriPure Isolation Reagent (Roche Applied Science, Mannheim, Germany), PrimeScript RT reagent Kit with gDNA Eraser (Perfect Real Time) (Toyobo, Osaka, Japan), and THUNDERBIRD SYBR qPCR Mix (Toyobo, Osaka, Japan), respectively, according to the manufacturer’s instructions. Hypoxanthine phosphoribosyltransferase 1 or β-actin was used as an internal control for the experiment with C57BL6J or *ob/ob* mice, respectively. The amplification of a single PCR product for each primer set was confirmed by melting curve analysis. Real-time PCR using the hypothalamus was performed with the annealing temperature at 63 °C. Each result was normalized to the average value of the control group and expressed as the relative mRNA level. The primer sequences are listed in Table 2. The Ob-R primers were designed to amplify all isoforms of Ob-R, whereas the transmembrane Ob-R primers specifically amplify isoforms containing the transmembrane domain.

### 2.4. Plasma Leptin Concentration

Plasma leptin concentration was measured using the Mouse/Rat Leptin ELISA Kit (Morinaga Institute of Biological Science, Inc., Yokohama, Kanagawa, Japan) according to the manufacturer’s instructions.

### 2.5. Plasma Soluble Leptin Receptor Concentration

Plasma sOb-R concentration was measured using a Mouse Leptin R DuoSet ELISA kit (R&D Systems, Minneapolis, MN, USA), according to the manufacturer’s instructions.

### 2.6. ADAM10 Activity

Hepatic ADAM10 activity was measured using a SensoLyte 520 ADAM10 Activity Assay Kit (AnaSpec Inc., Fremont, CA, USA), according to the manufacturer’s instructions.

### 2.7. FLI

The FLI was calculated using the plasma sOb-R and leptin concentrations, according to the following formula:

FLI = plasma leptin concentration/plasma soluble leptin receptor concentration

### 2.8. Statistical Analysis

To analyze the differences between the two groups, a Student’s *t*-test or a Welch’s *t*-test was performed for data with equal or unequal homogeneity of variance, respectively. To analyze the data composed of two factors, diet and leptin administration, a two-way analysis of variance was performed. The level of statistical significance was set at *p* < 0.05. All statistical analyses were performed using Excel 2016 (Bell Curve).

## 3. Results

### 3.1. Effect of Protein Restriction and Leptin Administration on Leptin and Ob-R in C57BL/6J Mice

**Figure 1:**
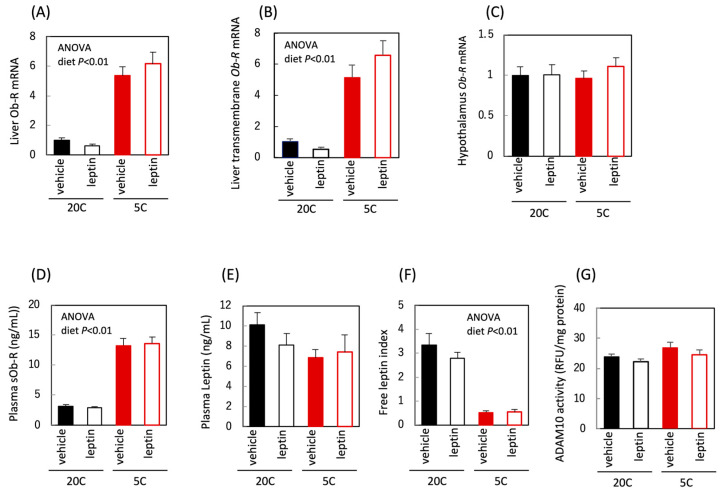
Effect of protein restriction and leptin administration on leptin and Ob-R in C57BL/6J mice. Liver total *Ob-R* mRNA (**A**), liver transmembrane *Ob-R* mRNA (**B**), hypothalamus *Ob-R* mRNA (**C**), plasma sOb-R concentration (**D**), plasma leptin concentration (**E**), plasma free leptin index (**F**), and liver ADAM10 activity (**G**) in C57BL/6J mice fed the control (20C) or the protein-restricted (5C) diet. mRNA levels were corrected for hypoxanthine *phosphoribosyltransferase 1* (*Hprt*) expression levels and expressed as a relative value to 20C vehicle group (**A**–**C**). All values are presented as the mean ± standard error of the mean (SEM) (*n* = 6 each group) (**A**–**G**). Two-way analysis of variance (ANOVA) was used to assess the main effects (diet and leptin administration), with *p* values indicated within the graphs.

We examined the effects of protein restriction and leptin administration on leptin and Ob-R levels in C57BL/6J mice. Total and transmembrane forms of *Ob-R* mRNA were increased (Figure 1A,B); however, total *Ob-R* mRNA was not affected by protein restriction in the hypothalamus (Figure 1C), demonstrating that the effect of protein restriction on Ob-R is liver-specific. Plasma sOb-R was increased by protein restriction, while plasma leptin was unchanged, resulting in a reduced plasma FLI (Figure 1D–F). Hepatic ADAM10 protease activity, which cleaves the extracellular region of Ob-R, remained unchanged following protein restriction or leptin administration (Figure 1G). These findings imply that protein restriction increased the synthesis of the transmembrane-type Ob-R in the liver, which was cleaved by ADAM10, resulting in an increase in circulating sOb-R in C57BL/6J mice.

### 3.2. Impact of Protein Restriction and Leptin Administration on Food Intake in C57BL/6J Mice

**Figure 2:**
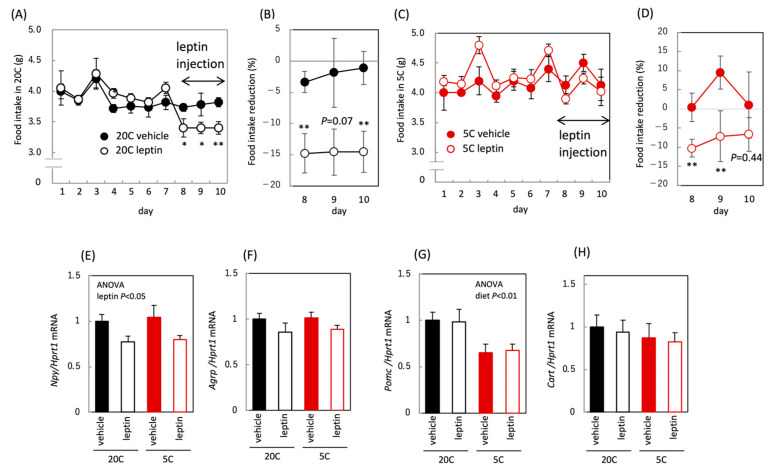
Effect of protein restriction and leptin administration on food intake in C57BL/6J mice. Food intake in C57BL/6J mice fed the control diet (20C) (**A**) or the protein-restricted diet (5C) (**C**), rate of food intake reduction by vehicle or leptin administration (**B**,**D**) and hypothalamus appetite-regulating peptide (*Npy*, *Agrp*, *Pomc*, *Cart*) mRNAs (**E**–**H**). Leptin was administered once daily for 4 days on days 8–11, and food intake was measured daily until day 10 (**A**–**D**). Percent reduction in daily food intake after vehicle or leptin administration relative to the pre-treatment mean (**B**,**D**). mRNA level was corrected by *Hprt* expression level and expressed as a relative value to 20C vehicle (**E**–**H**). All values are presented as the mean ± SEM (*n* = 6 each) (**A**–**H**). Significant differences are shown between the leptin-administered group and the vehicle-administered group (**A**–**D**); *, *p* < 0.05; **, *p* < 0.01. Two-way ANOVA was used to assess the main effects (diet and leptin administration), with *p* values indicated within the graphs (**E**–**H**).

To investigate the effects of leptin administration, we compared food intake between leptin-administered and non-administered groups of mice fed a control or low-protein diet. Food intake was reduced following leptin administration in both the control and the protein-restricted groups (Figure 2A–D), demonstrating that the appetite-reducing effect of leptin was not affected by protein restriction. In the hypothalamus, mRNA levels of appetite-regulating genes did not respond to leptin administration in either dietary group, with the exception of *Npy* (Figure 2E–H). Under the leptin administration conditions used in this study, it was difficult to detect leptin-induced changes in gene expression. In addition, Pomc expression was reduced by protein restriction, suggesting a regulatory shift toward increased appetite (Figure 2G).

### 3.3. Influence of Protein Restriction and Leptin Administration on Body and Adipose Tissue Weight in C57BL/6J Mice

**Figure 3:**
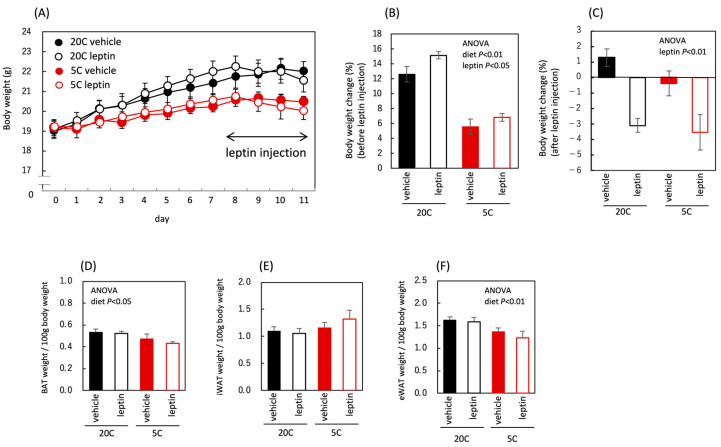
Influence of protein restriction and leptin administration on body and adipose tissue weight in C57BL/6J mice. Body weight (**A**), body weight change before and during vehicle or leptin treatment (**B**,**C**), and adipose tissue weight in C57BL/6J mice fed the control diet (20C) or the protein-restricted diet (5C) (**D**–**F**). Day 0 indicates the body weight on the day before feeding the experimental diet, and leptin was administered once daily for 4 days (days 8–11) (**A**). All values are presented as the mean ± SEM (*n* = 6 each) (**A**–**F**). Two-way ANOVA was used to assess the main effects (diet and leptin administration), with *p* values indicated within the graphs (**B**–**F**).

Body and adipose tissue weights were measured to verify the effects of protein restriction and leptin administration on body fat mass. Body weight gain was reduced by protein restriction before leptin administration, indicating that protein loss had occurred (Figure 3A,B). Leptin administration reduced body weight in both the control and protein-restricted groups (Figure 3A,C). Adipose tissue weights remained unchanged by leptin administration under the conditions of this experiment, whereas protein restriction reduced brown adipose tissue (BAT) and epididymal white adipose tissue (eWAT) weights (Figure 3D–F). Overall, the body and adipose tissue weights significantly decreased owing to protein restriction throughout the experimental period. Weight loss following leptin administration was observed in both diet groups.

### 3.4. Effect of Protein Restriction and Leptin Administration on Leptin and Ob-R in ob/ob Mice

**Figure 4:**
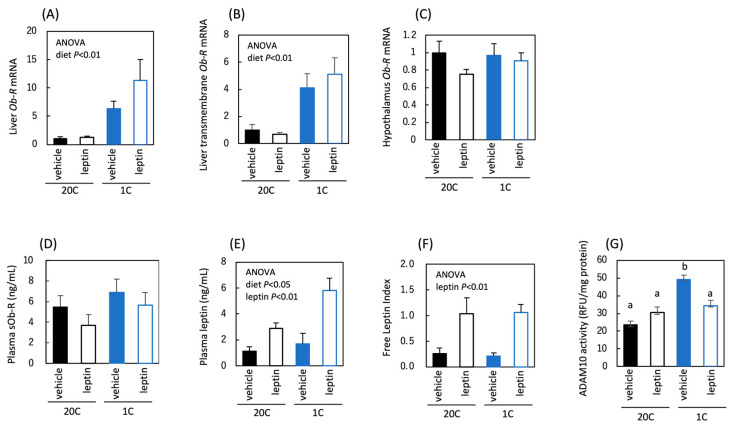
Effect of protein restriction and leptin administration on leptin and Ob-R in *ob/ob* mice. Liver total *Ob-R* mRNA (**A**), liver transmembrane *Ob-R* mRNA (**B**), hypothalamus *Ob-R* mRNA (**C**), plasma sOb-R (**D**), plasma leptin concentration (**E**), plasma free leptin index (**F**) and liver ADAM10 activity (**G**) in *ob/ob* mice fed the control (20C) or the protein-restricted (1C) diet. mRNA levels were corrected by *β-actin* expression level and expressed as a relative value to 20C vehicle (**A**–**C**). All values are presented as the mean ± SEM (*n* = 6 each) (**A**–**G**). Two-way ANOVA was used to assess the main effects (diet and leptin administration), with *p* values indicated within the graphs. When a significant interaction was detected, a multiple comparison test was performed (**G**); different letters (a, b) indicate significant differences among groups (*p* < 0.05).

We examined the effects of protein restriction and leptin administration on leptin and Ob-R levels in *ob/ob* mice. The total and transmembrane forms of *Ob-R* mRNA were increased by protein restriction in the liver (Figure 4A,B); however, *Ob-R* mRNA remained unchanged by protein restriction in the hypothalamus (Figure 4C). These changes in Ob-R were similar to those observed in C57BL/6J mice. However, conversely to C57BL/6J mice, plasma sOb-R levels were not altered by protein restriction in *ob/ob* mice (Figure 4D). Plasma leptin levels were increased by protein restriction and leptin administration, which was reflected in a higher FLI with leptin administration (Figure 4E,F). Protein restriction increased hepatic ADAM10 protease activity, which was higher in *ob/ob* mice than in C57BL/6J mice. Furthermore, unlike in C57BL/6J mice, the increase in ADAM10 activity was suppressed by leptin administration in *ob/ob* mice (Figure 4G). These findings indicate that *ob/ob* mice were similar to C57BL/6J mice in that protein restriction caused a liver-specific increase in *Ob-R* mRNA. In contrast, unlike in C57BL/6J mice, protein restriction did not increase plasma sOb-R levels in *ob/ob* mice. Hepatic ADAM10 activity in *ob/ob* mice was increased, or at least not decreased, upon protein restriction, suggesting that the lack of increase in circulating sOb-R was not due to reduced excision from the liver, but rather due to reduced stability of circulating sOb-R after shedding. In addition, the effect of leptin administration on plasma leptin levels was greater in *ob/ob* mice than in C57BL/6J mice, possibly because basal plasma leptin levels were lower in *ob/ob* mice.

### 3.5. Effect of Protein Restriction and Leptin Administration on Food Intake in ob/ob Mice

**Figure 5:**
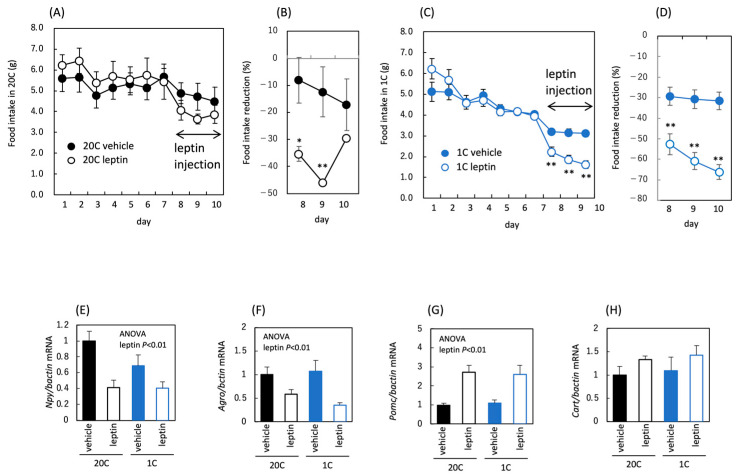
Impact of protein restriction and leptin administration on food intake in *ob/ob* mice. Food intake in *ob/ob* mice fed the control diet (20C) (**A**) or the protein-restricted diet (1C) (**C**), rate of food intake reduction by vehicle or leptin administration (**B**,**D**) and hypothalamus appetite-regulating peptide (*Npy*, *Agrp*, *Pomc*, *Cart*) mRNAs (**E**–**H**). Leptin was administered once daily for 4 days on days 8–11, and food intake was measured daily until day 10 (**A**–**D**). Percent reduction in daily food intake after vehicle or leptin administration relative to the pre-treatment mean (**B**,**D**). mRNA level was corrected by *β-actin* expression level and expressed as a relative value to 20C vehicle (**E**–**H**). All values are presented as the mean ± SEM (*n* = 6 each). Significant differences are shown between the leptin-administered group and the vehicle-administered group (**A**–**D**); *, *p* < 0.05, **, *p* < 0.01. Two-way ANOVA was used to assess the main effects (diet and leptin administration), with *p* values indicated within the graphs (**E**–**H**).

Food intake was reduced by leptin administration in both the control and the protein-restricted groups (Figure 5A–D), demonstrating that the appetite-reducing effect of leptin was not affected by protein restriction or sOb-R increase. The expression of genes involved in appetite regulation in the hypothalamus shifted toward appetite suppression in response to leptin administration in both dietary groups (Figure 5C–F). The effect of leptin on the expression of these genes was more apparent in the *ob/ob* mice than in the C57BL/6J mice. Considering that both appetite-lowering and appetite-related gene expression-modulating effects of administered leptin were observed in *ob/ob* mice, protein restriction did not suppress the effects of administered leptin in *ob/ob* mice.

### 3.6. Effect of Protein Restriction and Leptin Administration on Body and Adipose Tissue Weight in ob/ob Mice

**Figure 6:**
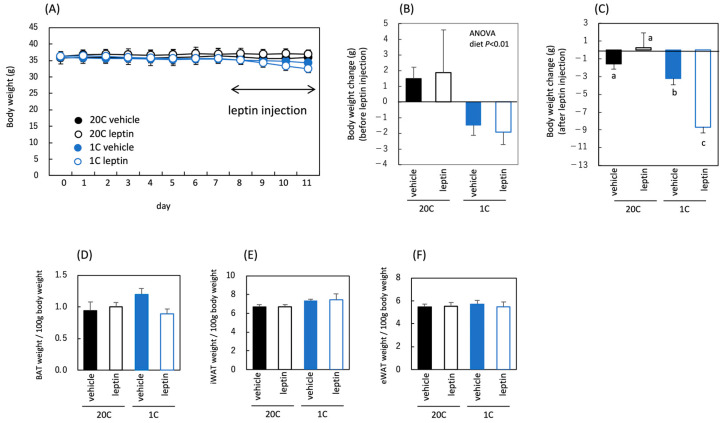
Effect of protein restriction and leptin administration on body and adipose tissue weight in *ob/ob* mice. Body weight (**A**), body weight change before and during vehicle or leptin treatment (**B**,**C**), and adipose tissue weight (**D**–**F**) in *ob/ob* mice fed the control diet (20C) or the protein-restricted diet (1C). Day 0 indicates the body weight on the day before feeding the experimental diet, and leptin was administered once daily for 4 days on days 8–11 (**A**). All values are presented as the mean ± SEM (*n* = 6 each). Two-way ANOVA was used to assess the main effects (diet and leptin administration), with *p* values indicated within the graphs (**B**–**F**). When a significant interaction was detected, a multiple comparison test was performed (**C**); different letters (a, b, c) indicate significant differences among groups (*p* < 0.05).

Body weight gain was reduced by protein restriction before leptin administration (Figure 6A,B) which was similar to that observed in C57BL/6J mice. Leptin administration reduced body weight only in the protein-restricted group, which reflected a marked decrease in food intake (Figure 6A,C). The adipose tissue weight per body weight was greater than that in C57BL/6J mice and was not affected by protein restriction or leptin administration (Figure 6D–F). Overall, the effects of protein restriction on body and adipose tissue weights were smaller in *ob/ob* than in C57BL/6J mice.

## 4. Discussion

In a previous study, we demonstrated that a low-protein diet increased hepatic *Ob-R* mRNA levels in C57BL/6J mice and that this increase was liver-specific and not observed in the hypothalamus [24]. Consistent with our previous findings, the expression of transmembrane *Ob-R* mRNA also increased during protein restriction in C57BL/6J mice in the present study, suggesting that the extracellular region of Ob-R synthesized in the liver was cleaved and released into the circulation as sOb-R. We further examined the effect of protein restriction on ADAM10, which has been reported to excise the extracellular domain of Ob-R in the liver [13]. ADAM10 is a transmembrane metalloproteinase that mediates the proteolytic shedding of various cell-surface molecules [33]. In C57BL/6J mice, plasma sOb-R increased despite no increase in ADAM10 activity under protein-restricted conditions. Therefore, the increase in circulating sOb-R concentrations in C57BL/6J mice during protein restriction is likely attributable primarily to enhanced hepatic Ob-R synthesis, with a relatively minor contribution from ADAM10 activity. In contrast, in ob/ob mice, hepatic ADAM10 activity increased under protein-restricted conditions; however, circulating sOb-R levels did not increase. The absence of an increase in circulating sOb-R in *ob/ob* mice may be attributable to the lack of leptin signaling; therefore, the impact of increased ADAM10 activity on circulating sOb-R concentrations in this model remains unclear. Our preliminary observations suggest that protein restriction induces only modest increases in Ob-R expression in extrahepatic tissues, including adipose tissue, compared with the liver, and that the marked increase in sOb-R is abolished in liver-specific Ob-R knockout mice, supporting the liver as the primary source of increased sOb-R.

In the present study, both C57BL/6J and *ob/ob* mice exhibited increased hepatic *Ob-R* mRNA levels under protein restriction. Several studies have examined the regulation of hepatic *Ob-R* mRNA expression. For instance, fasting, leptin stimulation, and decreased hepatic insulin signaling have been reported to increase *Ob-R* mRNA levels [21,23]. Although circulating insulin levels are low during protein restriction, hepatic insulin signaling is activated [29,30] and is therefore unlikely to contribute to the increase in *Ob-R* mRNA. Because the transcriptional regulation of the *Ob-R* gene downstream of insulin signaling remains unclear, it is possible that protein restriction selectively suppresses specific branches of insulin signaling, thereby increasing *Ob-R* mRNA expression. It has also been reported that the decrease in Ob-R expression associated with diet-induced obesity is mediated by C/EBP homologous protein (CHOP), which acts downstream of the endocannabinoid/cannabinoid CB1 receptor system [34]. Amino acid deficiency induces ATF4 activation; therefore, ATF4 may enhance Ob-R expression through CHOP activation. Nevertheless, the mechanism by which hepatic *Ob-R* mRNA levels increase during protein restriction remains unresolved.

In this study, we showed that protein restriction increased blood sOb-R levels in C57BL/6J mice but not in *ob/ob* mice. These results demonstrate that normal leptin is required for the increase in sOb-R levels under protein restriction. Our preliminary observations in *db/db* mice, another model lacking leptin signaling, indicate that the increase in circulating sOb-R during protein restriction is also abolished, supporting the requirement of intact leptin action for sOb-R elevation. One possible mechanism for the lack of increased blood sOb-R in *ob/ob* mice is its decreased half-life in the blood. Leptin in *ob/ob* mouse lacks the ability to bind to Ob-R, which may explain the inability of sOb-R to bind to leptin in the blood, resulting in a shortened half-life of sOb-R. Another possibility is that normal leptin activity is required to increase blood sOb-R levels under protein restriction. These possibilities appear inconsistent with the observation that leptin-treated *ob/ob* mice did not exhibit an increase in plasma sOb-R concentrations despite elevated circulating leptin levels. However, this discrepancy may be attributable to insufficient leptin action under the administration conditions used in this study. A more sustained and prolonged enhancement of leptin signaling, for example through continuous delivery via osmotic pumps, may be required to induce sOb-R elevation under protein-restricted conditions. It is also possible that obesity suppresses an increase in sOb-R levels in *ob/ob* mice. Lower circulating sOb-R levels in obese individuals support this possibility [18]. The effects of obesity on blood sOb-R levels under protein restriction are currently being investigated in our laboratory.

Due to the physiological significance of blood sOb-R, both the suppression and enhancement of leptin activity have been reported [14,15,17]. As sOb-R is also increased by fasting and the suppression of food intake [21,22], increased sOb-R is expected to play a role in adapting to malnutrition. The results of this study showed that the effects of exogenous leptin were not influenced by protein restriction in either C57BL/6J mice, in which serum sOb-R levels increased, or *ob/ob* mice, in which no such increase was observed. These findings suggest that an increase in circulating sOb-R under protein restriction may not regulate leptin action. On the other hand, increased sOb-R may also regulate leptin’s effects other than appetite suppression. A previous study has reported the function of sOb-R during food restriction. During restricted feeding, circulating sOb-R levels increase; however, this increase is absent in liver *Ob-R* knockout mice, which results in impaired thermogenesis from free fatty acids, reduced ability to maintain body temperature, and consequently increased mortality. These findings suggest that the elevation of sOb-R during food restriction serves a survival-promoting function. The increase in sOb-R observed during protein restriction may similarly have physiological significance, which warrants further investigation [35]. It has also been reported that sOb-R reduces the sepsis-enhancing effect of leptin by inhibiting leptin action [36]. Similar to the above-described possibility that decreased leptin action in the liver during protein restriction reduces the risk of liver inflammation, increased circulating sOb-R levels may reduce damage to multiple organs during infection. Regarding the physiological significance of sOb-R, which increases in response to protein restriction, we are currently conducting further research using liver-specific *Ob-R*-deficient mice.

In C57BL/6J mice, leptin administration did not significantly increase circulating leptin levels or reduce adipose tissue weight. However, the anorexigenic effect of leptin was confirmed, indicating that the administered leptin was biologically active. The lack of changes in circulating leptin levels and adipose tissue weight is likely attributable to the administration conditions. Specifically, circulating leptin levels may not have been elevated at the time of sampling due to the short half-life of administered leptin, and a longer duration of treatment may have been required to affect adipose tissue weight. *Ob/ob* mice are known to exhibit heightened sensitivity to exogenously administered leptin [37]. Consistent with this, in the present study, leptin administration induced more pronounced changes in the expression of appetite-regulating genes in the brains of *ob/ob* mice compared with C57BL/6J mice. Intracellular signaling pathways associated with appetite suppression were activated by leptin regardless of the presence or absence of protein restriction, leading to reduced food intake in both strains. Notably, because these intracellular signals were more robustly activated in *ob/ob* mice, the appetite-suppressing effect of leptin was also greater in this strain. In *ob/ob* mice, leptin administration increased circulating leptin levels but did not reduce adipose tissue weight. A longer duration of sustained leptin action may be required to decrease adipose tissue weight.

## 5. Conclusions

In conclusion, protein restriction increased circulating sOb-R levels in C57BL/6J mice but not in *ob/ob* mice, indicating that the upregulation of sOb-R requires endogenous leptin. The anorexigenic effect of exogenous leptin was unaffected by protein restriction in either strain. Although elevated circulating sOb-R during malnutrition has been hypothesized to modulate leptin action and contribute to adaptive responses, our findings demonstrate that the protein-restriction-induced increase in sOb-R does not influence leptin-induced appetite suppression. Further elucidation of the mechanisms responsible for the rise in circulating sOb-R during protein malnutrition, as well as its physiological significance, may provide new insights into leptin-mediated adaptations to nutritional stress. These findings provide a basis for future strategies to address malnutrition in children and the elderly.

## Figures and Tables

**Table 1:** Composition of the diet.

	20% Casein Diet (20C) (g/100 g Diet)	5% Casein Diet (5C) (g/100 g Diet)	1% Casein Diet (1C) (g/100 g Diet)
Casein	20	5	1
α-Cornstarch	43.45	53.61	56.32
Sucrose	21.73	26.81	28.16
L-Met	0.32	0.08	0.016
AIN93 vitamin mixture	1	1	1
AIN93G mineral mixture	3.5	3.5	3.5
Cellulose	5	5	5
Corn oil	5	5	5
Total	100	100	100

**Table 2:** Primer sequences used for real-time PCR.

		5′ → 3′
bactin	sense	AAGTGTGACGTTGACATCCGTAA
	antisense	GCAATGCCTGGGTACATGGT
HPRT1	sense	GCCGAGGATTTGGAAAAAGTG
	antisense	TTCATGACATCTCGAGCAAGTCTT
Ob-R	sense	TTGCTGCCTCTCTGCTGAAG
	antisense	GTCCTCCATGCCAATAGCAAA
transmembrane Ob-R	sense	TGGAAGGAGTTGGAAAACCAA
	antisense	TACAGCCCTGCGTCATTCTG
POMC	sense	TCCCCAGAGAGCTGCCTTT
	antisense	CCTGAGCGACTGTAGCAGAATCT
CART	sense	AGCTGATCGAAGCGTTGCA
	antisense	TTGGCCGTACTTCTTCTCGTAGA
AgRP	sense	GGCACAAGAGACCAGGACATC
	antisense	GAACACAACTCAGCAACATTGCA
NPY	sense	AGAAAACGCCCCCAGAACA
	antisense	TTGGAAAAGTCGGGAGAACAA

## Data Availability

The raw data supporting the conclusions of this article will be made available by the authors on request.

## Abbreviations

The following abbreviations are used in this manuscript:

ADAM 10	disintegrin and metalloproteinase domain 10
AgRP	agouti-related protein
BAT	brown adipose tissue
CART	cocaine- and amphetamine-regulated transcript
eWAT	epididymal white adipose tissue
FLI	free leptin index
iWAT	inguinal white adipose tissue
NPY	neuropeptide Y
Ob-R	leptin receptor
sOb-R	soluble leptin receptor
